# Optimal timing of surgical decompression for acute cervical spinal cord injury: a systematic review and network meta-analysis of randomized clinical trials

**DOI:** 10.3389/fneur.2026.1862411

**Published:** 2026-06-12

**Authors:** Yuzhou Chen, Yibo Dong, Longyun Yi, Hongbo Tu, Qiang Xu

**Affiliations:** 1 Department of Orthopedics, Wenjiang Traditional Chinese Medicine Hospital of Chengdu, Chengdu, China; 2 Department of Orthopedics, Chongqing Orthopedic Hospital of Traditional Chinese Medicine, Chongqing, China

**Keywords:** cervical spinal cord injury, network meta-analysis, neurological recovery, surgical decompression, surgical timing, ultra-early surgery

## Abstract

**Background:**

The optimal timing for surgical decompression in acute traumatic cervical spinal cord injury (SCI) remains a subject of intense clinical debate.

**Objective:**

To evaluate the comparative efficacy and safety of ultra-early (<24 h), early (<72 h), and delayed surgery using a network meta-analysis (NMA) framework.

**Methods:**

A systematic search of PubMed, Embase, and Web of Science identified randomized controlled trials (RCTs). Primary outcomes included American Spinal Injury Association (ASIA) Impairment Scale (AIS) improvement and ASIA motor score recovery. SUCRA (P-score) was used to rank treatment hierarchy.

**Results:**

Three RCTs involving 161 patients were included. Ultra-early surgery (<24 h) showed the highest clinical benefit trend for AIS improvement (OR: 2.08, 95% CI: 0.80–5.42) and motor recovery (MD: 11.22, 95% CI: −9.42 to 31.87). According to SUCRA, ultra-early surgery ranked first (68.8%), followed by early (61.5%) and delayed surgery (19.7%). Importantly, no significant increase in mortality (OR: 1.15) or complications (OR: 1.43) was observed with expedited intervention.

**Conclusion:**

Although limited by sample size, evidence suggests that ultra-early surgery within 24 h provides the best probability of neurological recovery without compromising patient safety.

**Systematic review registration:**

https://www.crd.york.ac.uk/PROSPERO/recorddashboard, CRD420261371811.

## 1 Introduction

Acute traumatic cervical spinal cord injury (SCI) is a devastating neurological event associated with profound morbidity, mortality, and socioeconomic burden (1). The pathophysiology of SCI involves a primary mechanical insult followed by a cascade of secondary injury mechanisms, including ischemia, edema, and cellular apoptosis. The widely recognized “time is spine” paradigm hypothesizes that expediting surgical decompression can mitigate these secondary cascades, thereby maximizing the potential for neurological recovery (2).

Over the past two decades, the definition of “early” surgery has continuously evolved. Earlier landmark randomized controlled trials (RCTs), such as the study by Vaccaro et al. (3), utilized a 72-h threshold but failed to demonstrate significant long-term clinical benefits compared to delayed surgery. Subsequently, large-scale prospective observational studies, most notably the Surgical Timing in Acute Spinal Cord Injury Study (STASCIS), shifted the paradigm by demonstrating improved outcomes with decompression within 24 h (“ultra-early” surgery) (4). Consequently, recent clinical guidelines increasingly advocate for ultra-early intervention (5). However, the majority of the current literature and previous meta-analyses rely heavily on observational cohort data, which are inherently susceptible to selection bias and confounding variables (2).

To date, high-level evidence exclusively from pure RCTs remains remarkably scarce (6), and direct head-to-head comparisons between the ultra-early (<24 h) and early (<72 h) surgical windows are practically nonexistent. Conventional pairwise meta-analyses are mathematically incapable of determining the relative superiority among multiple competing surgical timings. Therefore, this study aims to address this critical knowledge gap by conducting a network meta-analysis (NMA) of pure RCTs. By utilizing Surface Under the Cumulative Ranking curve (SUCRA) probabilities, we seek to establish a definitive evidence-based hierarchy of surgical timing to optimize outcomes for patients with acute cervical SCI.

## 2 Methods

### 2.1 Reporting methods and protocol

This systematic review and network meta-analysis (NMA) was prospectively registered on the PROSPERO database (Registration No. CRD420261371811). The study design, data synthesis, and final manuscript reporting strictly followed the guidelines set forth by the Preferred Reporting Items for Systematic Reviews and Meta-Analyses (PRISMA) and its extension for network meta-analyses (PRISMA-NMA).

### 2.2 Search strategy

A comprehensive and systematic literature search was conducted across multiple electronic databases, including PubMed, Embase, and the Web of Science, from their respective inception dates through April 2026. To minimize publication bias and capture ongoing or unpublished studies (grey literature), we additionally performed a thorough search of the clinical trial registries via ClinicalTrials.gov.

The search utilized a sophisticated combination of Medical Subject Headings (MeSH) terms and free-text keywords related to “cervical spinal cord injury,” “surgical decompression,” “early surgery,” “ultra-early surgery,” “surgical timing,” and “randomized controlled trials.” Boolean operators (“AND,” “OR”) were strategically applied to refine the search results. The search was restricted to human subjects and articles published in English. To ensure the reproducibility of our methodology, the complete and detailed search strings for each database are documented in Supplementary Table 1.

Furthermore, to ensure the inclusivity of the search, we manually screened the reference lists of all retrieved primary studies and previous systematic reviews or meta-analyses to identify any additional eligible trials that might have been overlooked by the initial electronic search. The study selection process followed the PRISMA (Preferred Reporting Items for Systematic Reviews and Meta-Analyses) guidelines to ensure transparency and methodological rigor.

### 2.3 Eligibility criteria

Studies were considered eligible for inclusion if they fulfilled the following pre-defined criteria based on the PICO (Population, Intervention, Comparison, Outcome) framework: Population: Adult patients (aged ≥18 years) diagnosed with acute traumatic cervical spinal cord injury (SCI), including those with or without pre-existing cervical spinal stenosis. Intervention: Surgical decompression performed within a specific early timeframe, defined as “ultra-early” (<24 h post-injury) or “early” (<72 h post-injury). Comparison: Delayed surgical intervention, typically defined as surgery performed later than 5 days or 2 weeks post-injury, or conservative treatment followed by elective surgery. Outcomes: At least one quantifiable neurological or safety outcome was reported, such as American Spinal Injury Association (ASIA) Impairment Scale (AIS) grade improvement, ASIA motor score recovery, or incidence of treatment-related complications and mortality. Study design: Only peer-reviewed, randomized controlled trials (RCTs) published in English were included to ensure the highest level of evidence.

### 2.4 Exclusion criteria

(1) non-randomized studies, including retrospective cohorts, case series, and observational studies; (2) studies involving non-traumatic SCI (e.g., tumors, infections, or transverse myelitis); (3) studies focusing on pediatric populations or patients with concomitant life-threatening systemic trauma; and (4) conference abstracts, editorials, and studies with insufficient data for meta-analysis extraction.

### 2.5 Quality assessment

Risk of bias was assessed using the Cochrane RoB 2 tool. Evidence certainty was graded according to the GRADE framework.

### 2.6 Statistical analysis

Pairwise and network meta-analyses were performed using the netmeta and meta packages in R (version 4.5.2). For dichotomous outcomes (AIS grade improvement, overall complications, and mortality), treatment effects were pooled using odds ratios (ORs) with corresponding 95% confidence intervals (CIs). For continuous outcomes (ASIA motor score recovery), mean differences (MDs) with 95% CIs were calculated. Statistical heterogeneity was evaluated using the *I^2^* statistic and *τ*^2^ variance, with *I^2^* > 50% considered indicative of substantial heterogeneity. A random-effects model was employed *a priori* to account for expected clinical and methodological heterogeneity across the included randomized controlled trials. Treatment hierarchy was evaluated using the Surface Under the Cumulative Ranking curve (SUCRA) probabilities (*p*-scores). Given the open V-shaped geometry of our network lacking closed loops, formal assessments of global and local inconsistency (e.g., node-splitting) were not applicable.

## 3 Results

### 3.1 Literature search and study selection

**Figure 1:**
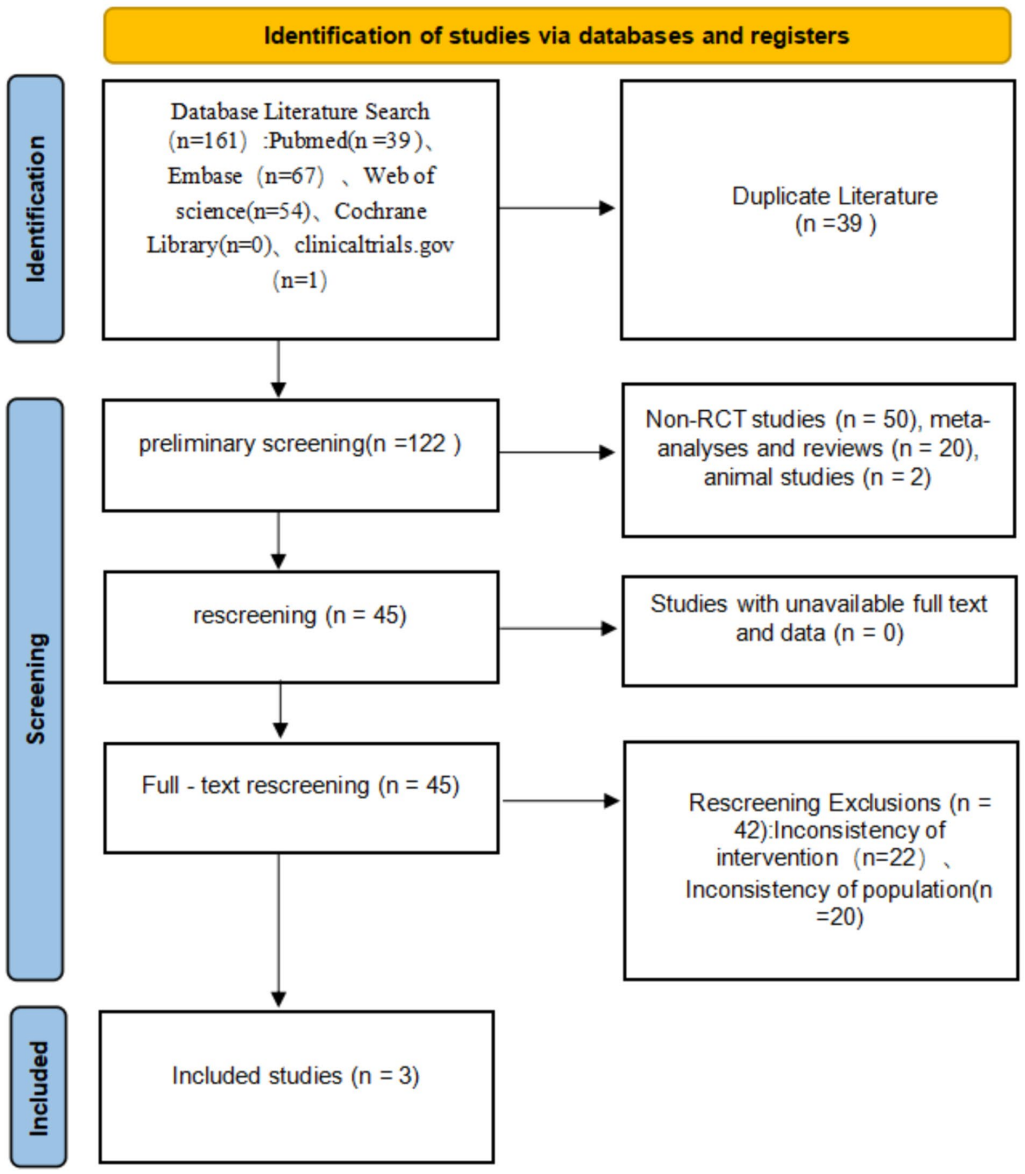
PRISMA 2020 flow diagram for new systematic reviews which included searches of databases and registers. Detailed flow diagram outlining the systematic literature search, screening, and selection process for eligible randomized controlled trials (RCTs). The chart quantifies the number of unique records identified from PubMed, Embase, Web of Science, and clinical trial registries, the reasons for exclusion during title and abstract screening, and the specific reasons for exclusion during full-text rescreening (e.g., inconsistency of intervention or population) to arrive at the final three included RCTs. *Consider, if feasible to do so, reporting the number of records identified from each database or register searched (rather than the total number across all databases/registers). **If automation tools were used, indicate how many records were excluded by a human and how many were excluded by automation tools. Source: Page MJ, et al. BMJ 2021; 372:n71. doi: 10.1136/bmj.n71. This work is licensed under CC BY 4.0. To view a copy of this license, visit https://creativecommons.org/licenses/.b.y/4.0/.

The initial systematic search across PubMed, Embase, and Web of Science yielded a total of 161 records (PubMed: 39; Embase: 67; Web of Science: 54; ClinicalTrials.gov: 1). After the removal of duplicates and a preliminary screening of titles and abstracts, 122 potentially eligible studies were retrieved for full-text evaluation. Following a rigorous application of the predefined inclusion and exclusion criteria, 3 high-quality, pure randomized controlled trials (RCTs) involving 161 participants were ultimately included in the qualitative and quantitative synthesis (see Figure 1).

### 3.2 Study characteristics and quality assessment

**Figure 2:**
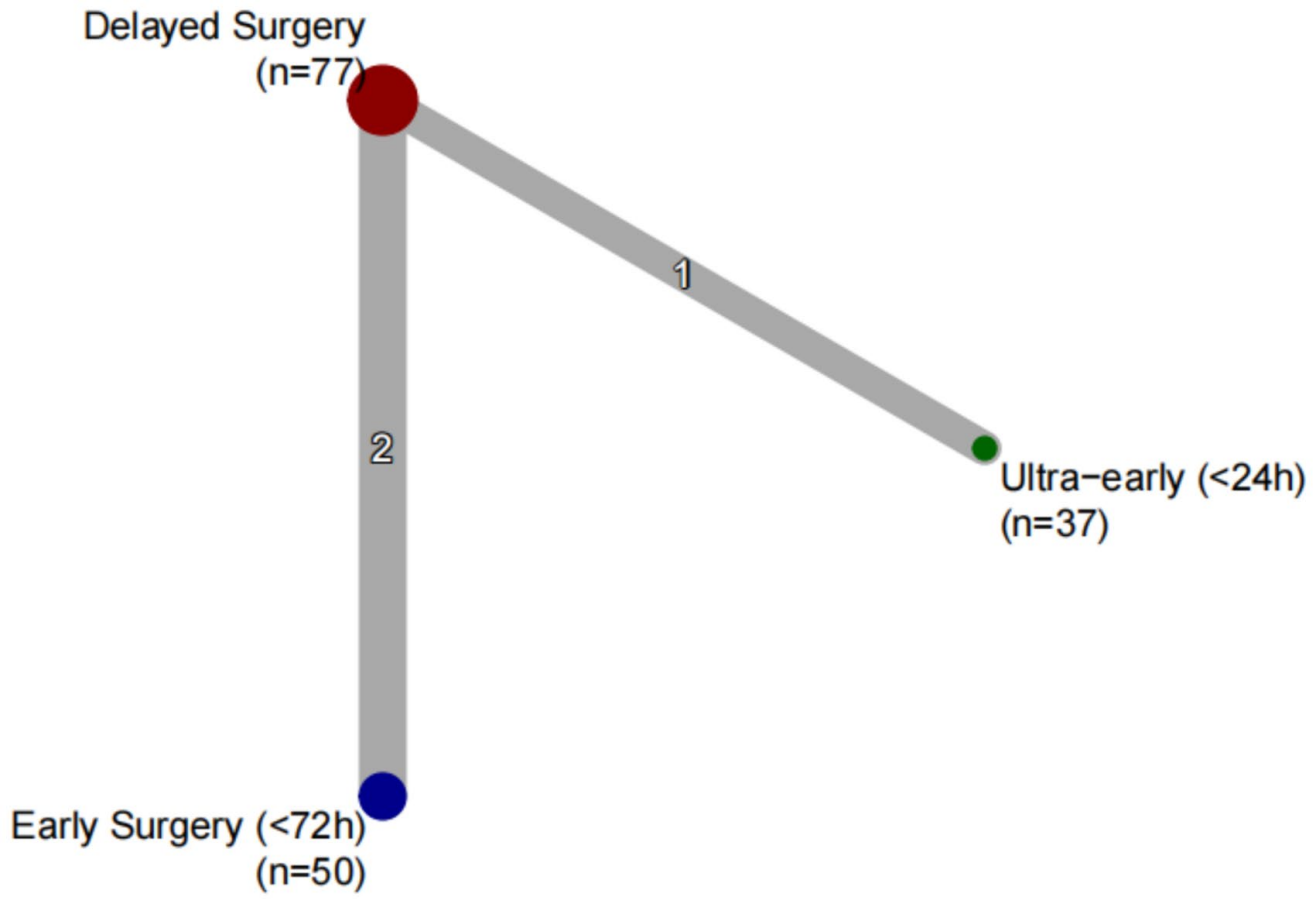
Network evidence plot. Visual representation of the network geometry for comparisons among ultra-early surgery (<24 h), early surgery (<72 h), and delayed surgery. The size of each node (circle) is proportional to the total sample size of patients assigned to that specific surgical window. The thickness of the connecting line (edge) and the numerical label correspond to the number of randomized controlled trials directly comparing the two connected interventions. The open V-shaped structure highlights the absence of direct head-to-head trials between ultra-early and early interventions, indicating that their relative efficacy relies on indirect comparisons bridged by the delayed surgery node.

The baseline characteristics of the included RCTs (3, 6, 7) are summarized in Table 1. The cumulative study population comprised 161 patients with acute traumatic cervical spinal cord injury. The network structure, illustrating the direct and indirect comparisons between ultra-early (<24 h), early (<72 h), and delayed surgery, is depicted in Figure 2.

**Table 1:** Characteristics of randomized controlled trials included in the meta-analysis.

Study (year)	Country	Study design	Sample size (E/D)	Timing definition (early vs. delayed)	Primary outcomes	Follow-up
Chikuda (6)	Japan	Multicenter RCT	37/33	<24 h vs. >2 weeks	ASIA motor score, SCIM	1 year
Xiao (7)	China	Single-center RCT	16/16	<72 h vs. >2 weeks	AIS grade, ASIA score	2 years
Vaccaro (3)	USA	Single-center RCT	34/28	<72 h vs. >5 days	ASIA motor score, LOS	Discharge

Baseline characteristics, study designs, and population distributions of the three included primary randomized controlled trials spanning from 1997 to 2021. Sample sizes are reported as the number of randomized patients in the early or ultra-early intervention group versus the delayed group (E/D). Primary and secondary outcomes extracted include raw neurological recovery scores and safety data across differing follow-up periods.

**Figure 3:**
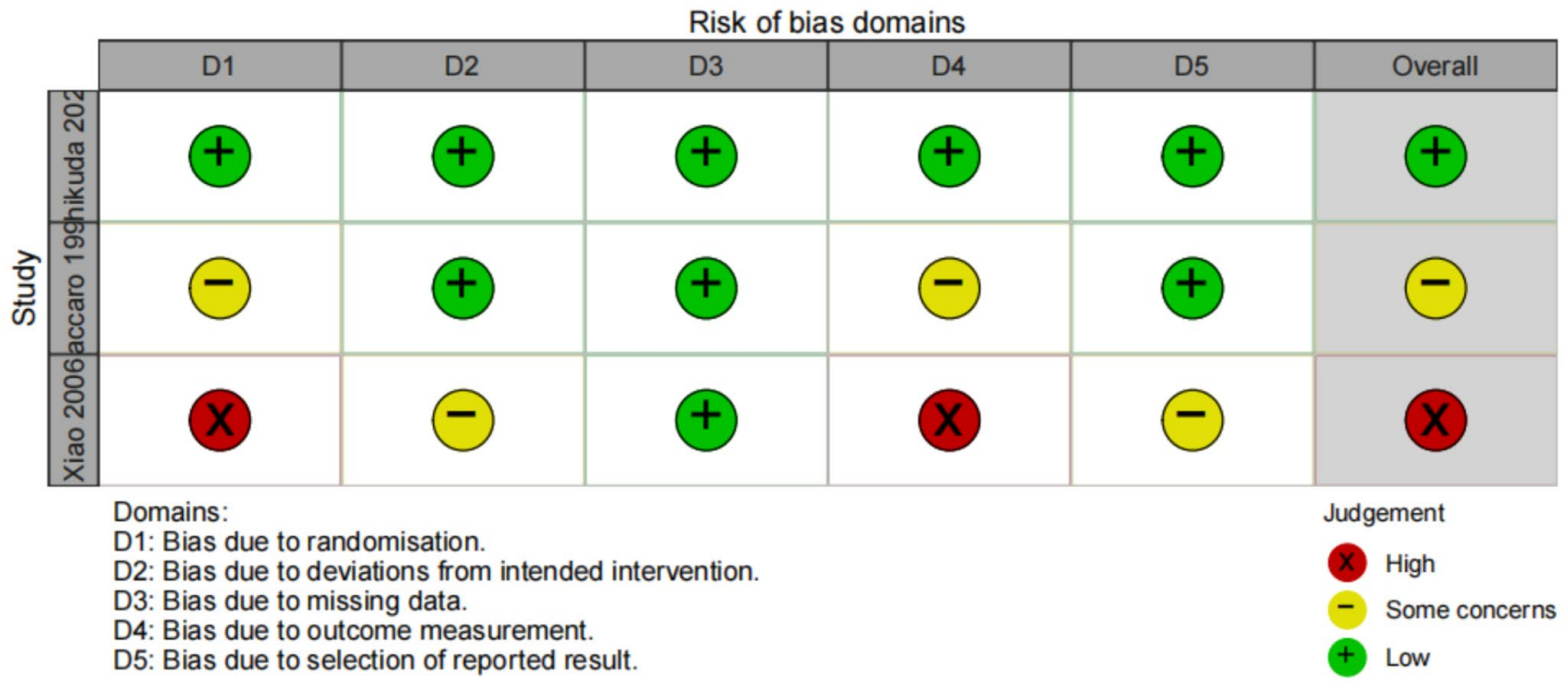
RoB 2 traffic light plot. Individual risk of bias assessment for each included randomized controlled trial using the Cochrane Risk of Bias 2 (RoB 2) tool for randomized trials. Symbols represent the risk level within each specific methodological domain: a green plus sign (+) indicates low risk of bias, a yellow minus sign (−) indicates some concerns, and a red cross sign (X) indicates high risk of bias. Individual domains evaluated include D1 (bias arising from the randomization process), D2 (bias due to deviations from intended interventions), D3 (bias due to missing outcome data), D4 (bias in measurement of the outcome), and D5 (bias in selection of the reported result), alongside an overall risk judgement.

**Figure 4:**
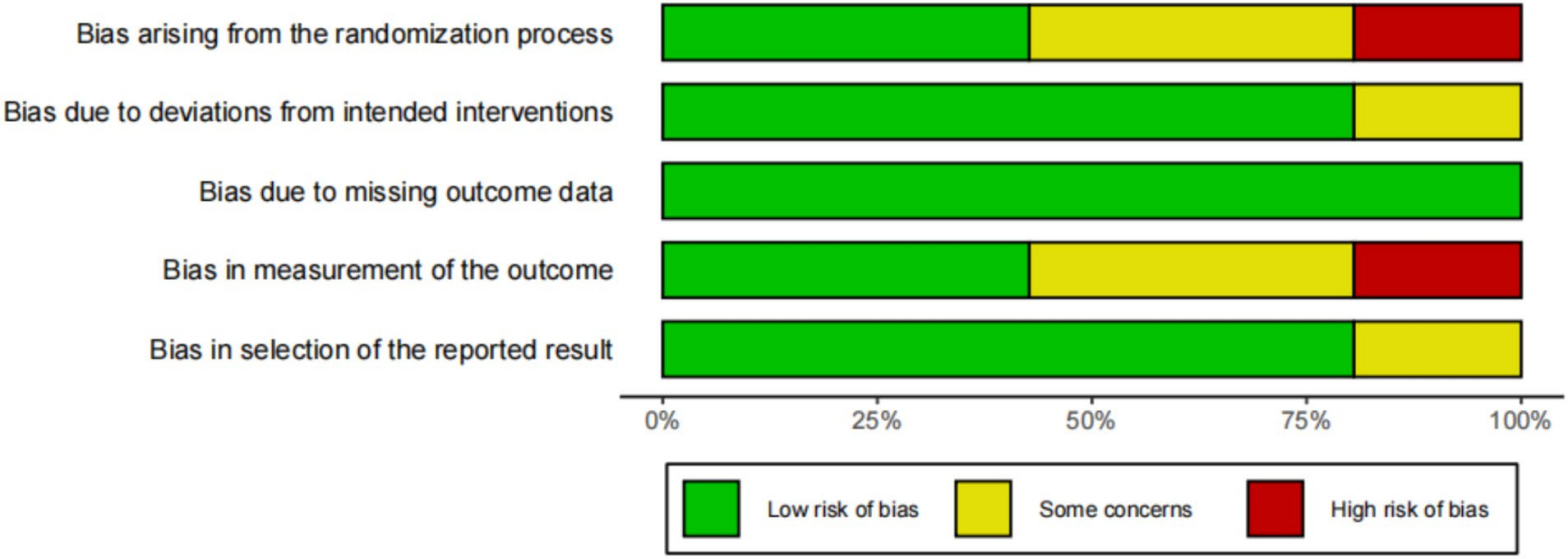
RoB 2 summary bar plot. Grouped bar chart summarizing the percentage distribution of risk of bias levels (low risk, some concerns, and high risk) across all included studies within each of the five Cochrane RoB 2 domains and the overall evaluation. Color-coded bars provide a high-level methodological synthesis, highlighting that blinding of outcome assessors and allocation concealment in emergency trauma environments represent the primary sources of potential bias in older trials.

Risk of bias assessment using the Cochrane RoB 2 tool revealed that the most recent trial (6) maintained a low risk across all domains. Conversely, the older trials (3, 7) exhibited “some concerns” or “high risk” primarily due to challenges in assessor blinding and allocation concealment in an emergency surgical setting (Figures 3, 4).

### 3.3 Primary outcome: AIS grade improvement

**Figure 5:**
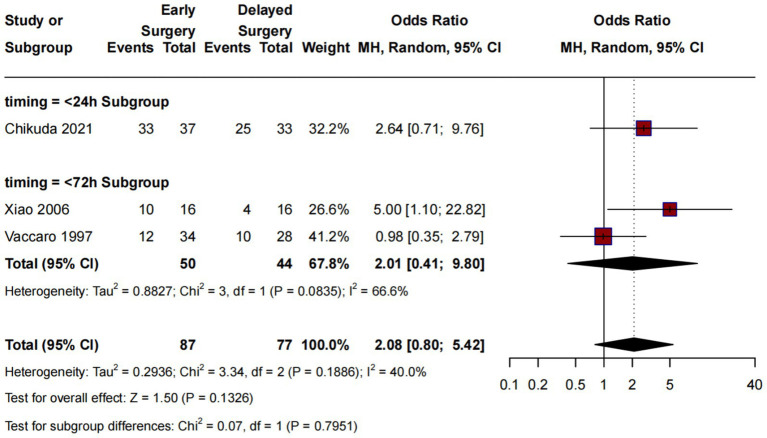
Forest plot for AIS improvement. Pairwise subgroup and pooled meta-analysis comparing ultra-early surgery (<24 h) and early surgery (<72 h) against delayed surgery for the primary binary outcome of American Spinal Injury Association Impairment Scale (AIS) grade improvement (≥1grade). Data were synthesized using a random-effects model under the Mantel–Haenszel (MH) method. Individual study effect sizes are represented by squares proportional to their statistical weight, with horizontal lines representing 95% Confidence Intervals (CIs). The pooled effect estimate for each subgroup and the overall population is depicted by the black diamonds. Statistical heterogeneity is quantified using the *I*^2^ statistic and *τ*^2^ (between-study variance).

The network meta-analysis revealed a favorable clinical trend for ultra-early intervention. Compared to delayed surgery, ultra-early decompression (<24 h) was associated with a higher likelihood of AIS grade improvement (OR 2.08, 95% CI 0.80–5.42). According to the GRADE framework, the certainty of evidence for this outcome was rated as Low (⊕ ⊕ ◯◯◯). The evidence was downgraded primarily due to “Serious” risk of bias (lack of assessor blinding in some trials) and “Serious” imprecision (total sample size failed to reach the optimal information size, with confidence intervals crossing the null line; see Figure 5).

### 3.4 Secondary outcome: ASIA motor score recovery

**Figure 6:**
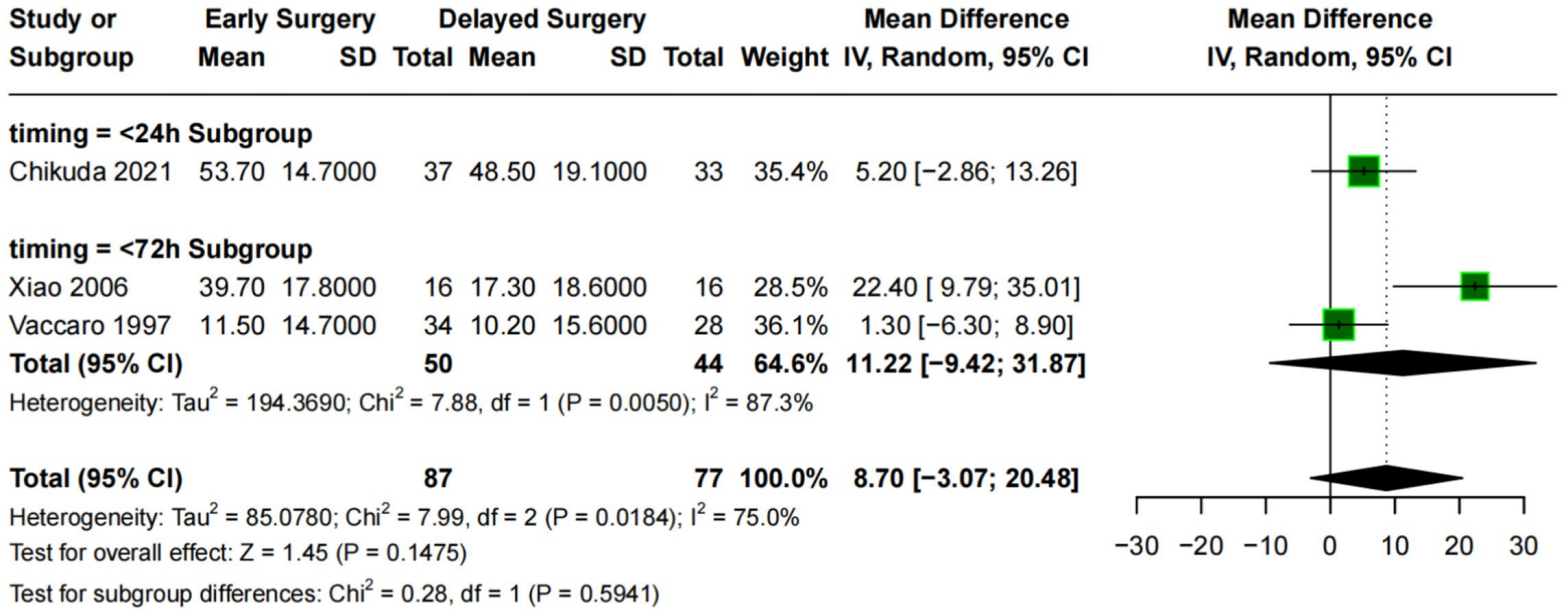
Forest plot for ASIA motor score recovery. Subgroup and cumulative pairwise meta-analysis evaluating the recovery of continuous American Spinal Injury Association (ASIA) motor scores among patients undergoing expedited versus delayed decompressive surgery. Pooled estimates were calculated using a random-effects model with the Inverse Variance (IV) method and expressed as Mean Differences (MDs) with 95% Confidence Intervals (CIs). Squares and horizontal lines indicate individual study means and CIs, while diamonds denote pooled estimates. Substantial statistical heterogeneity (*I*^2^ = 87.3%, *τ*^2^ = 194.3690) is observed within the <72 h subgroup, clinically driven by prominent variations in follow-up durations (ranging from discharge to 2 years) across historical trials.

Regarding functional recovery, ultra-early surgery showed a mean improvement of 11.22 points (95% CI -9.42 to 31.87) in ASIA motor scores compared to the delayed group. Similar to the AIS improvement, the GRADE certainty for motor recovery was assessed as Low (⊕ ⊕ ◯◯). Beyond the risk of bias, significant statistical heterogeneity (I^2^ = 87.3%) further necessitated a cautious interpretation of the pooled estimate, although this variance was clinically explained by differing follow-up durations across studies. Despite the high heterogeneity, pooling was deemed clinically justifiable under a random-effects model as all studies pointed towards the same direction of effect, though the magnitude of the pooled estimate should be interpreted with caution (see Figure 6).

### 3.5 Safety profile: overall complications and mortality

**Figure 7:**
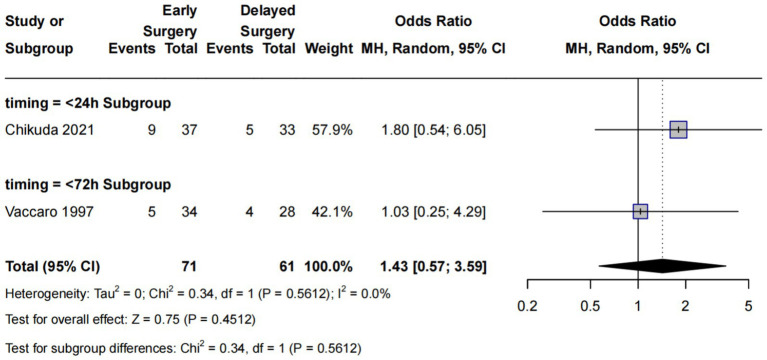
Forest plot for overall complications. Safety evaluation forest plot pooling the incidence of overall treatment-related severe complications between expedited (ultra-early and early) surgical decompression and delayed surgery groups. Statistical synthesis was performed using a random-effects model (Mantel–Haenszel method), with results presented as Odds Ratios (ORs) and 95% Confidence Intervals (CIs). The lack of statistically significant differences between subgroups (*p* = 0.56) indicates that expediting surgery does not compromise perioperative safety profiles.

**Figure 8:**
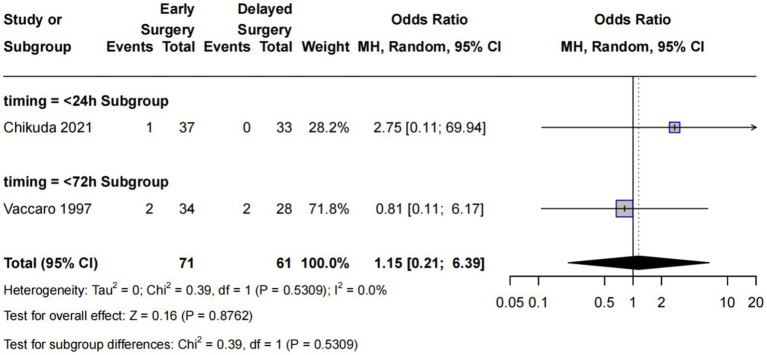
Forest plot for mortality. Safety forest plot analyzing all-cause mortality rates between early/ultra-early surgical decompression groups and delayed intervention cohorts. Pooled analysis under a random-effects model demonstrates a non-significant risk difference (OR 1.15, 95% CI 0.21–6.39). The exceptionally wide confidence intervals reflect the critical scarcity of clinical events and small sample sizes, demonstrating high statistical imprecision that warrants cautious interpretation.

Expedited surgery did not significantly increase the risk of overall complications (OR 1.43, 95% CI 0.57–3.59) or mortality (OR 1.15, 95% CI 0.21–6.39). The GRADE certainty for the safety profile was rated as Very Low (⊕◯◯◯). This further downgrading was driven by “Very Serious” imprecision, as the extremely low number of events and small cohort sizes resulted in exceptionally wide confidence intervals, precluding a definitive conclusion on relative safety risks (see Figures 7, 8).

### 3.6 Hierarchy of interventions (SUCRA ranking)

**Figure 9:**
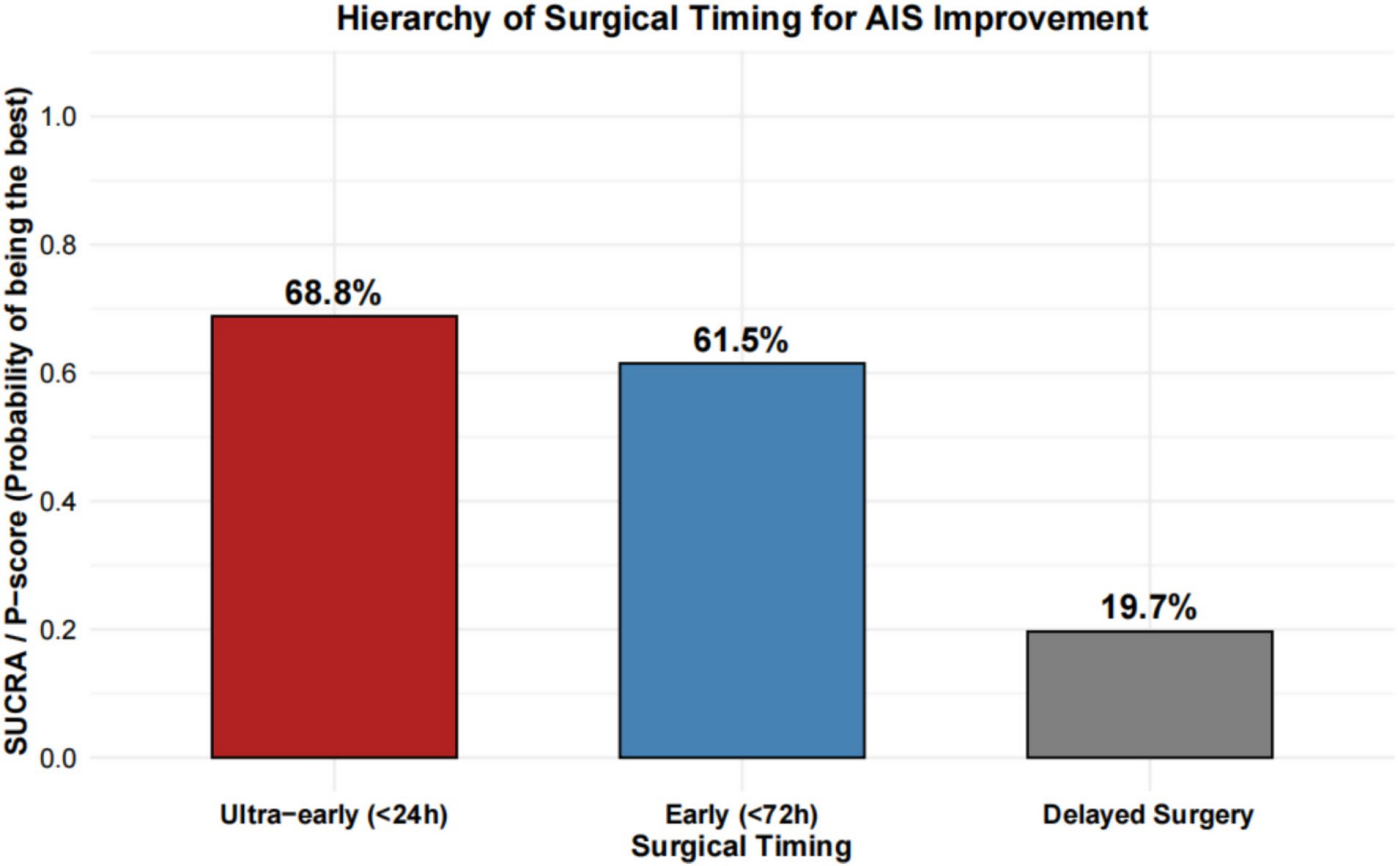
SUCRA ranking bar chart for AIS improvement. Hierarchy of surgical timings derived from network meta-analysis probabilities. Bars represent the Surface Under the Cumulative Ranking curve (SUCRA) scores (expressed as percentages), indicating the probability of each intervention being the optimal clinical strategy for maximizing AIS grade improvement. Higher SUCRA percentages correspond to a higher position in the treatment hierarchy. Ultra-early surgery within 24 h ranks first (68.8%), followed by early surgery within 72 h (61.5%) and delayed surgery (19.7%).

Despite the limitations in evidence certainty, SUCRA rankings consistently identified ultra-early surgery (<24 h) as the most probable optimal strategy for AIS improvement (68.8% probability). The comprehensive summary of findings, including the detailed GRADE assessments and reasons for downgrading, is presented in Table 2 and Figure 9.

**Table 2:** GRADE summary of findings.

Outcome indicator	No. of studies	Study design	Risk of bias	Inconsistency	Imprecision	Indirectness	Other consideration	Certainty
AIS improvement (≥1 grade)	3	randomised trials	serious^a^	not serious	serious^a^	not serious	None	⨁⨁◯◯ Low
ASIA motor score (mean difference)	3	randomised trials	serious^a^	not serious	serious^a^	not serious	None	⨁⨁◯◯ Low
Overall serious complications	2	randomised trials	serious^a^	not serious	very serious^b^	not serious	None	⨁◯◯◯ Very low

Structured evaluation of evidence certainty for each primary and secondary clinical outcome indicator determined via the Grading of Recommendations Assessment, Development and Evaluation (GRADE) software framework. Footnotes specify the explicit domain-specific justifications for downgrading evidence strength from high to low or very low certainty based on controlled clinical data.

## 4 Discussion

The present network meta-analysis of three randomized controlled trials (RCTs) represents the first comprehensive synthesis of high-level evidence specifically comparing ultra-early (<24 h), early (<72 h), and delayed surgical decompression for acute traumatic cervical SCI. Our primary finding indicates that while ultra-early intervention did not reach traditional statistical significance in pairwise comparisons, it demonstrated the highest probability of being the optimal strategy for both AIS grade improvement and motor score recovery, with a SUCRA value of 68.8%. This clinical trend supports the “time is spine” hypothesis, suggesting that earlier decompression may maximize the potential for neurological recovery. Importantly, this expedited surgical approach was not associated with an increased risk of mortality or overall complications (6, 7). While our analysis focused on the initial surgical window, it is increasingly recognized that long-term functional outcomes also depend on subsequent rehabilitation strategies, including advanced interventions like exoskeleton robotic training, which have shown promising results in improving mobility for SCI patients (8, 9).

The evolution of surgical timing in SCI has seen a paradigm shift over the past three decades, transitioning from a 72-h window initially proposed by Vaccaro et al. (3) to the 24-h “ultra-early” threshold advocated by the STASCIS study (4). While large-scale pooled analyses of individual patient data have recently corroborated the benefits of decompression within 24 h (10), these conclusions have predominantly relied on observational cohorts, which are inherently prone to selection bias and confounding. By restricting our analysis exclusively to pure RCTs, we minimize these biases and provide a more robust evaluation of the true therapeutic effect. We acknowledge the reviewer’s perspective that the contemporary definition of ‘ultra-early’ is progressively shifting towards narrower windows, such as <12 or even ≤8 h, to maximize neuroprotection. However, our 24-h threshold was strictly dictated by the pre-defined grouping criteria of the included foundational RCTs [e.g., Chikuda et al. (6)] and aligns with the landmark STASCIS protocol.

Furthermore, the persistent ambiguity regarding the definitions of “early” and “late” surgery across the literature—a challenge also highlighted by recent international multidisciplinary consensus groups for spinal cord compression (9, 11)—underscores the necessity of our hierarchical ranking to standardize the assessment of surgical windows. Although our study found no significant difference in overall safety, clinicians must remain vigilant regarding specific morbidities; for instance, recent evidence identifies age and injury severity as critical predictors of post-traumatic dysphagia, a common but often overlooked complication in acute cervical SCI patients (12).

Recently, several high-quality studies have further explored surgical timing paradigms in acute cervical SCI. For instance, an updated systematic review by Fehlings et al. (13) and a meta-analysis by Nazwar et al. (14) have corroborated the broader benefits of expedited care on functional trajectories. More specifically, Yu et al. (15) demonstrated that while intervention within 24 h reduces complications and hospital stays, pushing the surgical window to “ultra-early” (≤8 h) significantly maximizes neurological recovery, as evidenced by ASIA score improvements. Concurrently, Vasquez-Paredes et al. (16) reinforced these findings, showing that early surgical decompression within 24 h yields better neurological outcomes and reduces postoperative complications, particularly in patients with complete SCI.

While these contemporary studies contribute highly robust, large-scale evidence supporting expedited care, many incorporate mixed data sources, including observational cohorts or propensity-matched registries. The added value of our current network meta-analysis lies in strictly isolating high-level evidence exclusively from pure RCTs. By eliminating the inherent selection biases found in observational cohorts and utilizing an NMA framework, our study provides a mathematically rigorous treatment hierarchy. This demonstrates that the clinical trends observed in recent real-world and mixed-method systematic reviews are fundamentally corroborated by controlled trial environments, solidifying the ultra-early (<24 h) window as the most probable optimal intervention.

A critical observation in our analysis is the divergence between clinical magnitude and statistical significance regarding ultra-early decompression. Although ultra-early surgery (<24 h) demonstrated a robust clinical trend with an odds ratio (OR) of 2.08 for AIS improvement, the result did not reach the conventional threshold of statistical significance (*p* > 0.05). This phenomenon is likely attributable to a Type II error, driven by the small cumulative sample size (*n* = 161) inherent in pure RCTs of acute SCI. As emphasized in recent spine research methodology, relying solely on frequentist *p*-values can be misleading, especially in rare surgical conditions where the “Fragility Index” is typically high (17, 18). The SUCRA ranking (68.8% for <24 h) and the magnitude of the effect size provide a more nuanced and clinically relevant interpretation of the “time is spine” hierarchy than a binary *p*-value alone (19). Furthermore, while traditional RCTs remain the gold standard, the growing role of propensity score methods and real-world data (RWD) in spine research underscores that clinical trends observed in small-sample trials often align with outcomes from larger, high-quality observational registries (20, 21). Therefore, the non-significant *p*-value in our primary outcome should be interpreted as a reflection of limited statistical power rather than a lack of therapeutic efficacy. In the present network meta-analysis, the random-effects model was employed as the primary analytical framework to account for both within-study sampling error and between-study variability. Unlike the fixed-effect model, which assumes a single true effect size across all included trials, the random-effects approach acknowledges the inherent clinical and methodological heterogeneity within the field of spinal cord injury (SCI) surgery (22). Given the notable discrepancies in surgical techniques, patient demographics (ranging from young trauma victims to elderly patients with stenosis), and follow-up durations among the included RCTs, the random-effects model provides a more conservative and generalizable estimate of the therapeutic effect (23, 24). This methodological choice ensures that the pooled estimates are not disproportionately driven by any single large-scale trial, thereby enhancing the robustness of our hierarchical ranking of surgical timings (25, 26).

From a clinical safety perspective, historical reluctance to perform ultra-early surgery often stemmed from concerns over exacerbating hemodynamic instability or perioperative morbidity in polytrauma patients. However, our pooled safety profiles unequivocally demonstrate that expedited decompression within 24 h does not increase the risk of mortality (OR 1.15) or overall severe complications (OR 1.43). This confirmed safety allows clinicians to safely capitalize on the profound pathophysiological benefits of early decompression. At the microenvironmental level, the primary mechanical insult triggers a devastating secondary injury cascade characterized by progressive tissue edema, elevated intraspinal pressure (ISP), and subsequent local ischemia. Recent experimental evidence underscores that promptly alleviating ISP through surgical interventions (such as duraplasty or bony decompression) is crucial for halting edema expansion and preventing irreversible axonal loss (27, 28). Furthermore, prolonged cord compression drastically compromises local hemodynamics. As highlighted by recent multi-center clinical trials and neurocritical care cohorts, actively maintaining spinal cord perfusion pressure (SCPP)—either through timely surgical decompression or adjunctive cerebrospinal fluid (CSF) drainage—is paramount to mitigating ischemic damage in the acute phase (29, 30). Without timely intervention, the resulting severe hypoxic microenvironment not only drives cellular apoptosis but also hinders spontaneous neuroregeneration, creating a hostile milieu that emerging neuroprotective therapies, such as hypoxia-conditioned mesenchymal stem cells, are now being designed to combat (31, 32). Therefore, expeditious surgical unroofing of the spinal canal acts as the definitive first step to break this vicious cycle of ischemia and swelling, laying the vital physiological foundation for both natural recovery and future biologic adjuncts.

## 5 Limitations

Despite the rigorous methodology and inclusion of the highest level of evidence, several limitations inherent to this study must be acknowledged.

First, the most prominent constraint is the limited number of eligible randomized controlled trials (*n* = 3) and the relatively small cumulative sample size. This resulted in ‘imprecision’ within the GRADE assessment, leading to wide confidence intervals that cross the line of no effect for several outcomes. Consequently, the statistical power to detect subtle differences between ultra-early and early intervention was constrained, although the SUCRA rankings provided a clear hierarchical trend.

Second, the network geometry is characterized by a V-shaped structure lacking a closed loop. Because no direct head-to-head RCTs exist comparing ultra-early (<24 h) and early (<72 h) surgery, our findings regarding their relative efficacy rely entirely on indirect comparisons bridged by the delayed surgery group. While NMA methodology is robust for such inferences, the absence of direct evidence necessitates a cautious interpretation of the rankings.

Third, we observed substantial statistical heterogeneity in ASIA motor score recovery (*I*^2^ = 87.3%). This variance is likely driven by clinical discrepancies across trials, including differences in patient demographics (e.g., elderly patients with pre-existing stenosis vs. young high-energy trauma victims) and drastically different follow-up durations.

Lastly, our search was restricted to peer-reviewed articles published in English, which may have excluded relevant trials published in other languages, potentially introducing a degree of language bias. Strictly speaking, our network forms an open V-shape rather than a closed loop, relying entirely on indirect comparisons between <24 h and <72 h via the delayed surgery node. Therefore, the SUCRA rankings should be interpreted as exploratory rather than definitive.

Nevertheless, given the immense ethical and logistical challenges of conducting pure RCTs in the emergency surgical setting of SCI, this study represents the most comprehensive and up-to-date synthesis of the highest-quality evidence currently available to guide clinical decision-making.

## 6 Conclusion

In conclusion, this systematic review and network meta-analysis of pure randomized controlled trials suggests that surgical decompression within 24 h of acute traumatic cervical spinal cord injury represents the optimal therapeutic window for maximizing neurological and motor recovery. Although the certainty of evidence for primary outcomes is currently rated as low to very low according to the GRADE framework—primarily due to imprecision driven by small cumulative sample sizes—the hierarchical SUCRA rankings consistently favor ultra-early intervention (<24 h, 68.8% probability) over early (<72 h) and delayed surgery. Importantly, expediting surgical decompression does not increase the risk of mortality or overall complications, confirming its safety profile in hemodynamically stable patients. Given the catastrophic nature of spinal cord injury and the “time is spine” paradigm, ultra-early intervention within 24 h represents a reasonable and potentially optimal strategy in clinical practice. Further large-scale, high-quality multicenter RCTs are warranted to strengthen the evidence base and refine these surgical timing recommendations.

## Data Availability

The original contributions presented in the study are included in the article/Supplementary material, further inquiries can be directed to the corresponding author.
