# Supplementary material for: Optimal timing of surgical decompression for acute cervical spinal cord injury: a systematic review and network meta-analysis of randomized clinical trials
Source: Front Neurol. 2026 Jun 12;17:1862411. doi: 10.3389/fneur.2026.1862411 (PMC13303211; doi:10.3389/fneur.2026.1862411)
Supplement: Supplementary file 1 [file Supplementary_file_1.doc]

Supplementary Table 1: Detailed Search Strategies for Electronic Databases

(Date of Search: April 2026)

1. PubMed (MEDLINE)

Note: The search strategy incorporates Medical Subject Headings (MeSH) and text words [tw] / title/abstract [tiab]. The highly sensitive Cochrane filter for identifying randomized trials was applied.

(("Spinal Cord Injuries"[MeSH] OR "Cervical Cord"[MeSH] OR "Trauma, Nervous System"[MeSH]) OR (cervical spinal cord injur*[tiab] OR cervical cord injur*[tiab] OR cervical spine injur*[tiab] OR tetraplegia[tiab] OR quadriplegia[tiab] OR SCI[tiab])) AND (("Decompression, Surgical"[MeSH] OR "Orthopedic Procedures"[MeSH] OR "Neurosurgical Procedures"[MeSH]) OR (surg*[tiab] OR decompress*[tiab] OR operat*[tiab] OR laminectomy[tiab] OR discectomy[tiab])) AND (("Time-to-Treatment"[MeSH] OR "Time Factors"[MeSH]) OR (tim*[tiab] OR early[tiab] OR ultra-early[tiab] OR delayed[tiab] OR acute[tiab] OR hour*[tiab] OR optimal timing[tiab])) AND ((randomized controlled trial[pt] OR controlled clinical trial[pt] OR randomized[tiab] OR placebo[tiab] OR clinical trials as topic[mesh:noexp] OR randomly[tiab] OR trial[ti]) NOT (animals[mh] NOT humans[mh]))

2. Embase

Note: The search utilizes Emtree terms (/exp) and title/abstract keywords (:ab,ti). A validated RCT filter for Embase was applied.

(('spinal cord injury'/exp OR 'cervical spinal cord'/exp OR ('cervical spinal cord injur*' OR 'cervical cord injur*' OR 'cervical spine injur*' OR tetraplegia OR quadriplegia):ab,ti) AND ('decompression surgery'/exp OR 'orthopedic surgery'/exp OR 'neurosurgery'/exp OR (surg* OR decompress* OR operat* OR laminectomy OR discectomy):ab,ti) AND ('time to treatment'/exp OR 'time factor'/exp OR (tim* OR early OR 'ultra-early' OR delayed OR acute OR hour*):ab,ti) AND ('randomized controlled trial'/exp OR 'controlled clinical trial'/exp OR random*:ab,ti OR placebo:ab,ti))

3. Web of Science (Core Collection)

Note: The search relies on Topic (TS) fields encompassing title, abstract, author keywords, and Keywords Plus.

TS=("spinal cord injur*" OR "cervical cord injur*" OR "cervical spine injur*" OR tetraplegia OR quadriplegia) AND TS=(surg* OR decompress* OR operat* OR laminectomy OR discectomy) AND TS=(tim* OR early OR "ultra-early" OR delayed OR acute OR hour* OR window) AND TS=(randomized OR randomised OR randomly OR placebo OR trial OR RCT)

4. ClinicalTrials.gov

Note: Advanced search function used to identify registered, ongoing, or unpublished trials to minimize publication bias.

(Cervical Spinal Cord Injury) AND (Surgery OR Decompression) AND (Early OR Timing) filters:Interventional
